# Supplementary figures and images for: Response of salt stress resistance in highland barley (Hordeum vulgare L. var. nudum) through phenylpropane metabolic pathway
Source: PLoS One. 2023 Oct 3;18(10):e0286957. doi: 10.1371/journal.pone.0286957 (PMC10547159; doi:10.1371/journal.pone.0286957)

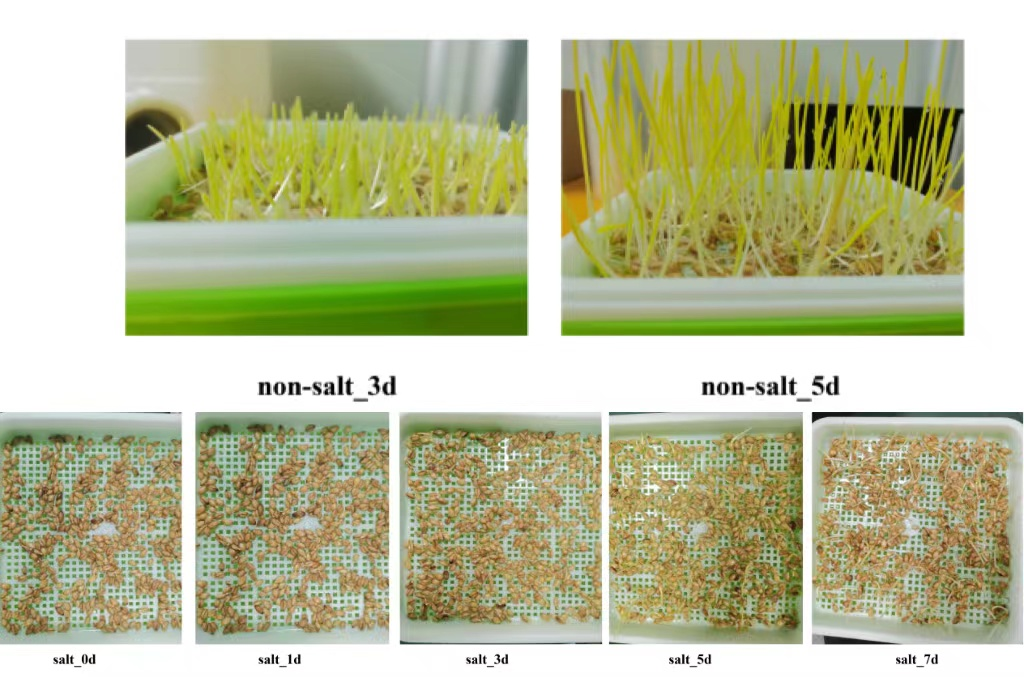

Supplement: S1 Fig — (TIF) [file pone.0286957.s001.tif]

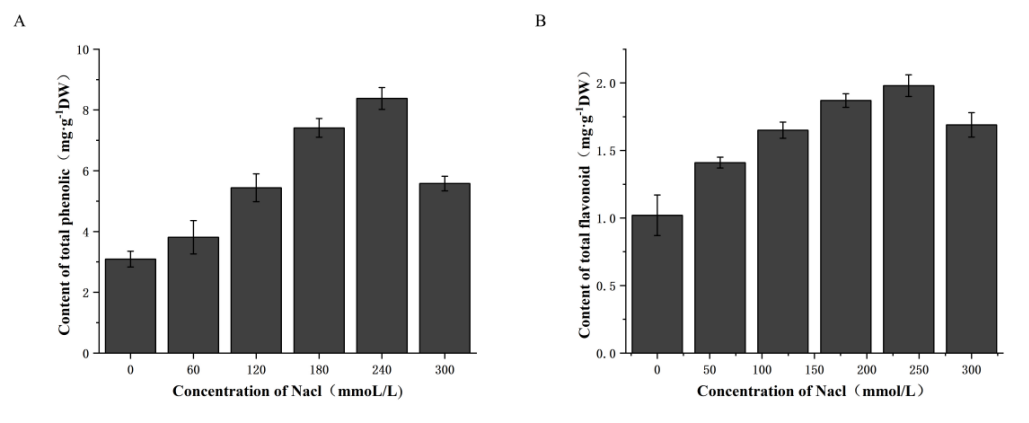

Supplement: S2 Fig — (TIF) [file pone.0286957.s002.tif]

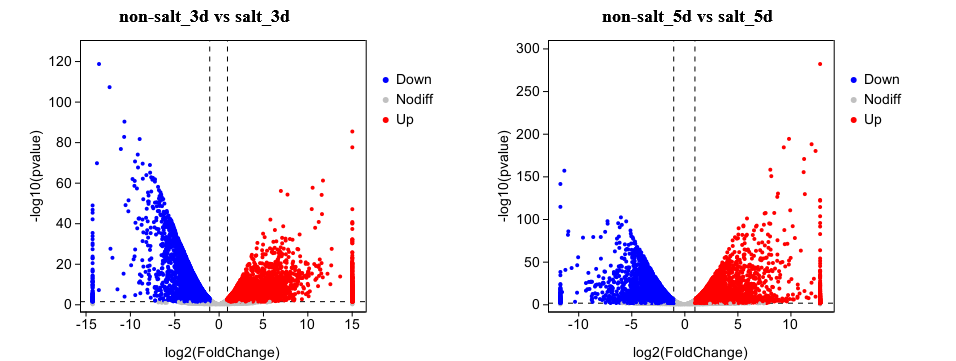

Supplement: S3 Fig — (TIF) [file pone.0286957.s003.tif]

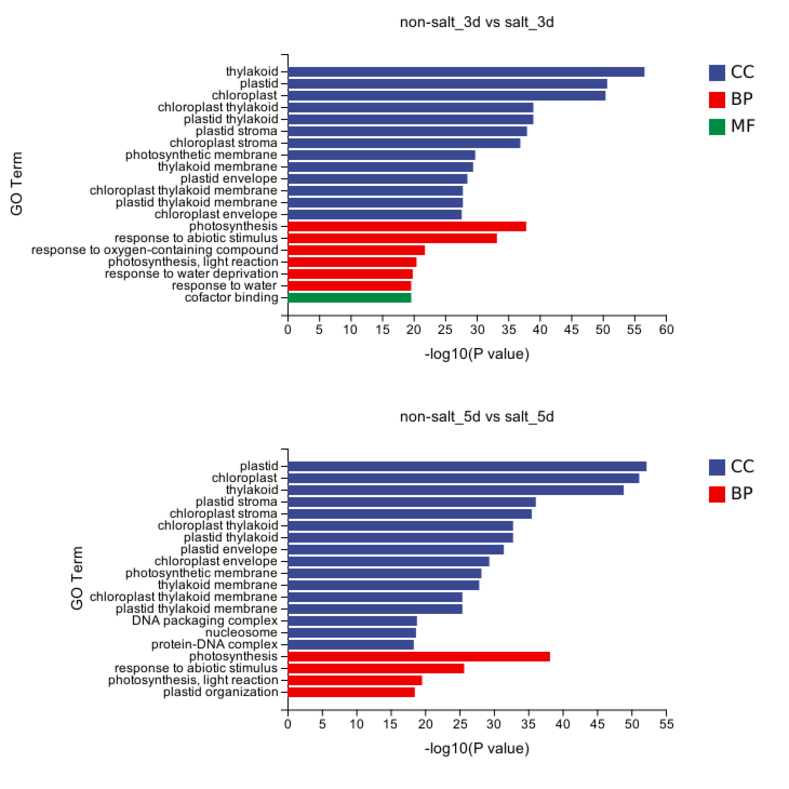

Supplement: S4 Fig — (TIF) [file pone.0286957.s004.tif]

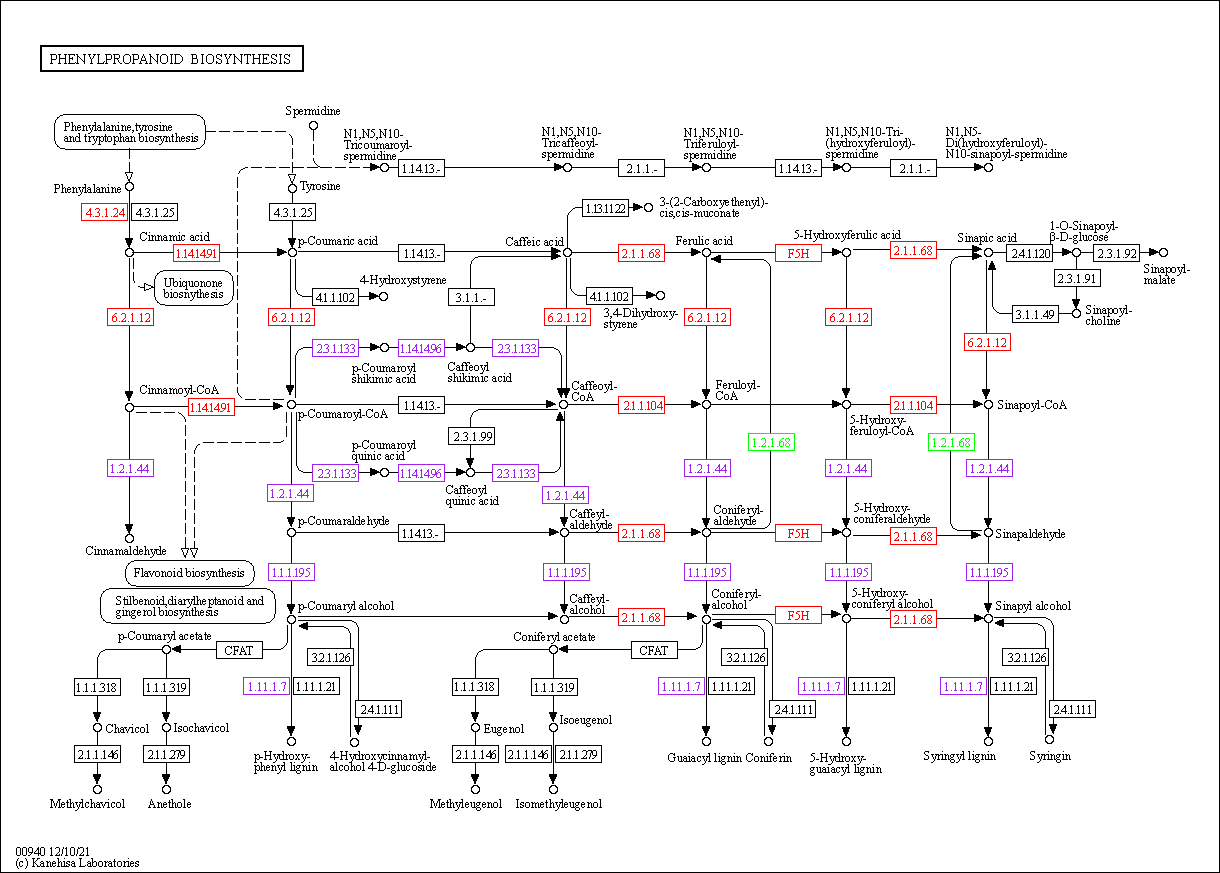

Supplement: S5 Fig — (TIF) [file pone.0286957.s005.tif]
