# Supplementary material for: Response of salt stress resistance in highland barley (Hordeum vulgare L. var. nudum) through phenylpropane metabolic pathway
Source: PLoS One. 2023 Oct 3;18(10):e0286957. doi: 10.1371/journal.pone.0286957 (PMC10547159; doi:10.1371/journal.pone.0286957)
Supplement: S1 Table — (DOCX) [file pone.0286957.s006.docx]

**Table S1.** The content of antioxidants

| Day | Content of total phenolic（mg·g^-1^DW） | | Content of total flavonoid（mg·g^-1^DW） | | Content of total ferulic acid（μg·g^-1^DW） | | Content of total caffeic acid（μg·g^-1^DW） | |
| --- | --- | --- | --- | --- | --- | --- | --- | --- |
|  | non-salt | salt | non-salt | salt | non-salt | salt | non-salt | salt |
| 0d | 2.15±0.25d | 3.46±0.26e | 0.98±0.11c | 1.01±0.01d | 2.01±0.14e | 2.35±0.05e | 0.21±0.034e | 0.26±0.06e |
| 1d | 2.76±0.21b | 5.53±0.47d | 1.01±0.13c | 1.05±0.02d | 3.83±0.12c | 7.31±0.06c | 1.26±0.15d | 1.61±0.19d |
| 3d | 3.09±0.26a | 8.38±0.36a | 1.62±0.15a | 1.98±0.08a | 8.97±0.06a | 12.22±0.35d | 2.89±0.16a | 4.69±0.24a |
| 5d | 2.31±0.14c | 7.97±0.23b | 1.20±0.11b | 1.48±0.04b | 4.59±0.14b | 8.53±0.23b | 1.98±0.14b | 2.71±0.14b |
| 7d | 2.19±0.18cd | 7.38±0.28c | 1.02±0.09c | 1.20±0.05c | 2.94±0.1d | 4.34±0.32d | 1.42±0.07c | 2.12±0.05c |
