# Supplementary material for: Response of salt stress resistance in highland barley (Hordeum vulgare L. var. nudum) through phenylpropane metabolic pathway
Source: PLoS One. 2023 Oct 3;18(10):e0286957. doi: 10.1371/journal.pone.0286957 (PMC10547159; doi:10.1371/journal.pone.0286957)
Supplement: S2 Table — (DOCX) [file pone.0286957.s007.docx]

| Sample | Raw_Read_Number | Raw_Bases | Trimmed_Read_Number | Raw_N_rate | Raw_Q30_rate |
| --- | --- | --- | --- | --- | --- |
| salt_0d_1 | 58068304 | 8710245600 | 52894790 | 0.00013 | 93.4 |
| salt_0d_2 | 48425046 | 7263756900 | 44565396 | 0.000134 | 93.31 |
| salt_0d_3 | 47727498 | 7159124700 | 44098368 | 0.000128 | 93.31 |
| salt_d1_1 | 42081098 | 6312164700 | 38645336 | 0.000127 | 93.76 |
| salt_d1_2 | 46755688 | 7013353200 | 43050090 | 0.000129 | 93.6 |
| salt_d1_3 | 43210744 | 6481611600 | 39541796 | 0.000127 | 93.99 |
| salt_d3_1 | 42122866 | 6318429900 | 38851034 | 0.000127 | 93.81 |
| salt_d3_2 | 40056332 | 6008449800 | 36818362 | 0.000125 | 94.02 |
| salt_d3_3 | 48050146 | 7207521900 | 43953506 | 0.000131 | 93.99 |
| salt_d5_1 | 51665316 | 7749797400 | 47017090 | 0.00013 | 93.75 |
| salt_d5_2 | 41639986 | 6245997900 | 38787592 | 0.000127 | 93.89 |
| salt_d5_3 | 42176360 | 6326454000 | 38922324 | 0.000115 | 91.93 |
| salt_d7_1 | 44220290 | 6633043500 | 40686170 | 0.000127 | 93.65 |
| salt_d7_2 | 39369158 | 5905373700 | 36465976 | 0.000126 | 93.64 |
| salt_d7_3 | 41460090 | 6219013500 | 38362548 | 0.000128 | 93.36 |
| Non-salt_d3_1 | 48151120 | 7270819120 | 45916088 | 0.000674 | 94.83 |
| Non-salt_d3_2 | 52375152 | 7908647952 | 49866818 | 0.000677 | 95.25 |
| Non-salt_d3_3 | 51812412 | 7823674212 | 49400390 | 0.001338 | 94.51 |
| Non-salt_d5_1 | 43380824 | 6550504424 | 41304800 | 0.000718 | 95.15 |
| Non-salt_d5_2 | 48716826 | 7356240726 | 46276430 | 0.000671 | 94.71 |
| Non-salt_d5_3 | 45491396 | 6869200796 | 43304610 | 0.000678 | 94.64 |

**Table S2.** Characteristics of libraries
