# Supplementary material for: Response of salt stress resistance in highland barley (Hordeum vulgare L. var. nudum) through phenylpropane metabolic pathway
Source: PLoS One. 2023 Oct 3;18(10):e0286957. doi: 10.1371/journal.pone.0286957 (PMC10547159; doi:10.1371/journal.pone.0286957)
Supplement: S3 Table — (DOCX) [file pone.0286957.s008.docx]

**Table S3.** Genes altered significantly in transcription level

| **Pathway: phenylpropane metabolic pathway** |
| --- |
| **Non-salt_3d vs salt_3d** |
| Up-regulated genes: D1007_21721;D1007_26284;D1007_35264;D1007_22042;D1007_12720;D1007_12710;D1007_45845;D1007_55042;D1007_19603;D1007_23020;D1007_62265;D1007_35647;D1007_62039;D1007_45713;D1007_20297;D1007_20254;D1007_08519;D1007_13934;D1007_02829;D1007_03378;D1007_12702;D1007_56767;D1007_12721;D1007_58836;D1007_20251;D1007_08523;D1007_20384;D1007_02548;D1007_16120;D1007_59786;D1007_22362;D1007_11280;D1007_60001;D1007_39853;D1007_53752;D1007_32152;D1007_06590;D1007_15902;D1007_56490;D1007_55051;D1007_59709;D1007_49825;D1007_12676;D1007_50682;D1007_60648;D1007_29110;D1007_23170D1007_34900;D1007_05762;D1007_04302;D1007_01228;D1007_15922;D1007_60670;D1007_12705;D1007_48397;D1007_24083;D1007_32446;D1007_30726;D1007_53440;D1007_27295;D1007_53754;D1007_46850;D1007_18702;D1007_16393;D1007_27800;D1007_35632;D1007_07135;D1007_28609;D1007_35368;D1007_55070;D1007_26794;D1007_41496;D1007_34306;D1007_24049;D1007_31839;D1007_08336;D1007_58538;D1007_12424;D1007_44018;D1007_53787;D1007_46587;D1007_50878;D1007_59479;D1007_56448;D1007_44149;D1007_23096;D1007_12709;D1007_23334;D1007_36610;D1007_24282;D1007_42212;D1007_35123;D1007_12723;D1007_61074;D1007_38922;D1007_44884;D1007_35630;D1007_59577;D1007_11287;D1007_50681;D1007_32332;D1007_30699;D1007_50689;D1007_24864;D1007_27617;D1007_12708;D1007_12965;D1007_31768;D1007_60664;D1007_56495;D1007_24931;D1007_60660;D1007_35129;D1007_47620;D1007_24209;D1007_59788;D1007_55052;D1007_40068;D1007_41509;D1007_44505;D1007_56451;D1007_49083 |
| Down-regulated genes:  D1007_42761;D1007_24084;D1007_46099;D1007_46097;D1007_54290;D1007_55035;D1007_23152;D1007_34916;D1007_41753;D1007_28076;D1007_22043;D1007_45711;D1007_45705;D1007_44327;D1007_56492;D1007_55047;D1007_24043;D1007_53663;D1007_24285;D1007_22592;D1007_09337;D1007_05552;D1007_21785;D1007_56443;D1007_58172;D1007_14600;D1007_08086;D1007_37657;D1007_11466;D1007_49219;D1007_12180;D1007_12699;D1007_02186;D1007_52014;D1007_33116;D1007_51183;D1007_13556;D1007_51067;D1007_60974;D1007_02872;D1007_23363;D1007_40828;D1007_37159;D1007_05467;D1007_48439;D1007_23150;D1007_39201;D1007_46096 |

| **Pathway: phenylpropane metabolic pathway** |
| --- |
| **Non-salt_3d vs salt_3d** |
| Up-regulated genes: D1007_21721;D1007_26284;D1007_35264;D1007_22042;D1007_12720;D1007_12710;D1007_45845;D1007_55042;D1007_19603;D1007_23020;D1007_62265;D1007_35647;D1007_62039;D1007_45713;D1007_20297;D1007_20254;D1007_08519;D1007_13934;D1007_02829;D1007_03378;D1007_12702;D1007_56767;D1007_12721;D1007_58836;D1007_20251;D1007_08523;D1007_20384;D1007_02548;D1007_16120;D1007_59786;D1007_22362;D1007_11280;D1007_60001;D1007_39853;D1007_53752;D1007_32152;D1007_06590;D1007_15902;D1007_56490;D1007_55051;D1007_59709;D1007_49825;D1007_12676;D1007_50682;D1007_60648;D1007_29110;D1007_23170D1007_34900;D1007_05762;D1007_04302;D1007_01228;D1007_15922;D1007_60670;D1007_12705;D1007_48397;D1007_24083;D1007_32446;D1007_30726;D1007_53440;D1007_27295;D1007_53754;D1007_46850;D1007_18702;D1007_16393;D1007_27800;D1007_35632;D1007_07135;D1007_28609;D1007_35368;D1007_55070;D1007_26794;D1007_41496;D1007_34306;D1007_24049;D1007_31839;D1007_08336;D1007_58538;D1007_12424;D1007_44018;D1007_53787;D1007_46587;D1007_50878;D1007_59479;D1007_56448;D1007_44149;D1007_23096;D1007_12709;D1007_23334;D1007_36610;D1007_24282;D1007_42212;D1007_35123;D1007_12723;D1007_61074;D1007_38922;D1007_44884;D1007_35630;D1007_59577;D1007_11287;D1007_50681;D1007_32332;D1007_30699;D1007_50689;D1007_24864;D1007_27617;D1007_12708;D1007_12965;D1007_31768;D1007_60664;D1007_56495;D1007_24931;D1007_60660;D1007_35129;D1007_47620;D1007_24209;D1007_59788;D1007_55052;D1007_40068;D1007_41509;D1007_44505;D1007_56451;D1007_49083 |
| Down-regulated genes:  D1007_42761;D1007_24084;D1007_46099;D1007_46097;D1007_54290;D1007_55035;D1007_23152;D1007_34916;D1007_41753;D1007_28076;D1007_22043;D1007_45711;D1007_45705;D1007_44327;D1007_56492;D1007_55047;D1007_24043;D1007_53663;D1007_24285;D1007_22592;D1007_09337;D1007_05552;D1007_21785;D1007_56443;D1007_58172;D1007_14600;D1007_08086;D1007_37657;D1007_11466;D1007_49219;D1007_12180;D1007_12699;D1007_02186;D1007_52014;D1007_33116;D1007_51183;D1007_13556;D1007_51067;D1007_60974;D1007_02872;D1007_23363;D1007_40828;D1007_37159;D1007_05467;D1007_48439;D1007_23150;D1007_39201;D1007_46096 |

| **Pathway：Citrate cycle (TCA cycle)** |
| --- |
| **Non-salt_3d vs salt_3d** |
| Up-regulated genes:  D1007_40561;D1007_19329;D1007_03290 |
| Down-regulated genes:  D1007_56275;D1007_19354;D1007_17832 |
