# Supplementary material for: Response of salt stress resistance in highland barley (Hordeum vulgare L. var. nudum) through phenylpropane metabolic pathway
Source: PLoS One. 2023 Oct 3;18(10):e0286957. doi: 10.1371/journal.pone.0286957 (PMC10547159; doi:10.1371/journal.pone.0286957)
Supplement: S4 Table — (DOCX) [file pone.0286957.s009.docx]

| Key enzyme | Gene ID | Non-salt_3d vs salt_3d (log_2_FoldChange) |
| --- | --- | --- |
| PAL | D1007_11280 | 4.945 |
|  | D1007_08519 | 2.638 |
|  | D1007_46587 | 2.308 |
| 4CL | D1007_26794 | Inf |
|  | D1007_39853 | 4.515 |
| CH4 | D1007_58538 | 3.003 |
| CAD | D1007_31191 | 0.285 |
|  | D1007_24864 | 1.536 |
|  | D1007_23170 | 4.215 |
| COMT | D1007_07135 | 7.088 |
|  | D1007_60001 | 11.209 |
|  | D1007_22362 | 6.709 |
|  | D1007_08336 | 2.239 |
|  | D1007_58836 | 3.818 |
| CCoAOMT | D1007_03378 | 5.364 |
| CCR | D1007_34900 | 5.420 |
|  | D1007_56767 | 4.563 |
|  | D1007_35632 | 3.062 |
|  | D1007_35630 | 1.369 |
| REF1 | D1007_13556 | -1.269 |

**Table S4.** Transcriptional level analysis of key genes in the phenylpropane metabolic pathway
