# Supplementary material for: Response of salt stress resistance in highland barley (Hordeum vulgare L. var. nudum) through phenylpropane metabolic pathway
Source: PLoS One. 2023 Oct 3;18(10):e0286957. doi: 10.1371/journal.pone.0286957 (PMC10547159; doi:10.1371/journal.pone.0286957)
Supplement: S5 Table — (DOCX) [file pone.0286957.s010.docx]

**Table S5.** Primers used for qRT-PCR

| Key enzyme | Gene ID | Primers | Sequence (5’-3’) |
| --- | --- | --- | --- |
| PAL | D1007_08519 | Forward primer | GCCGAGGCCATTGACATATT |
|  |  | Reversed primer | CAGCTCTTGACAGCGTTCTT |
|  | D1007_11280 | Forward primer | CTCGCGTCCATTGTTCTCTT |
|  |  | Reversed primer | TGTACTCTGGCTTGCCATTC |
| CH4 | D1007_58538 | Forward primer | CAAGAAGGCGTTGGAGAAGA |
|  |  | Reversed primer | ATAGGACGTTGTCGTGGTTG |
| 4CL | D1007_39853 | Forward primer | CCCATGTTCCACGTCTACTC |
|  |  | Reversed primer | ACCAGCTCCATCATCTTGAC |
| COMT | D1007_58836 | Forward primer | ACAACCTTGAGGGTGGAAAG |
|  |  | Reversed primer | GAAGATGTAGGTGGAACGGAAG |
| CCoAOMT | D1007_35630 | Forward primer | CACCATCCTCCGCAAGTT |
|  |  | Reversed primer | CGCTGGTTCGACATCTTGTA |
| CCR | D1007_03378 | Forward primer | GTTCGACTTCGCGTTCGT |
|  |  | Reversed primer | GTGTTGTCGTAGGCGATGAG |
|  | D1007_34900 | Forward primer | GGAGCGACTACGACTACTGC |
|  |  | Reversed primer | CACAAGCATGCACGATGACG |
| CAD | D1007_24864 | Forward primer | TCAGCTCGTCCAACAAGAAG |
|  |  | Reversed primer | CACGGTGTCGATGATGTAGTC |
| GAPDH  (housekeeping gene) | D1007_16168 | Forward primer | GTGGTGTCAACGAGAAGGAATA |
|  |  | Reversed primer | GGTCCACACCTTAGCAAGAG |
