# Supplementary material for: Response of salt stress resistance in highland barley (Hordeum vulgare L. var. nudum) through phenylpropane metabolic pathway
Source: PLoS One. 2023 Oct 3;18(10):e0286957. doi: 10.1371/journal.pone.0286957 (PMC10547159; doi:10.1371/journal.pone.0286957)
Supplement: Attachment — Submitted filename: PONE-D-23-04873 reviewed.pdf [file pone.0286957.s011.pdf]

# PLOS ONE

## Response of stress resistance in highland barley ( Hordeum vulgare L. var. nudum ) through phenylpropane metabolic pathway

### --Manuscript Draft--

| | |
|---|---|
| Manuscript Number: | PONE-D-23-04873 |
| Article Type: | Research Article |
| Full Title: | Response of stress resistance in highland barley ( Hordeum vulgare L. var. nudum ) through phenylpropane metabolic pathway |
| Short Title: | Response of stress resistance in highland barley through phenylpropane metabolic pathway |
| Corresponding Author: | Ming Zhao<br>Anhui Polytechnic University<br>Wuhu, Anhui CHINA |
| Keywords: | Highland barley; salt stress; transcriptome; Phenylpropane metabolic pathway. |
| Abstract: | Highland barley (Hordeum vulgare L. var. nudum) is a kind of grain crop, which grows on the plateau under poor and high salt conditions. Therefore, to cultivate high-quality highland barley varieties, it is necessary to study the molecular mechanism of strong resistance in highland barley, which has not been clearly explained.<br>In this study, high concentration of NaCl (240 mmol/L), simulating the unfavorable environment, was used to spray to the treated highland barley seeds. Transcriptomic analysis revealed that the expression of more than 8,000 genes in highland barley seed cells was significantly altered, suggesting that the metabolic landscape of the cells was deeply changed under salt stress. Through the KEGG analysis, the phenylpropane metabolic pathway was significantly up-regulated under salt stress, resulting in the accumulation of polyphenols, flavonoids, and lignin, the metabolites for improving the stress resistance of highland barley seed cells, being increased 2.71, 1.22, and 1.17 times, respectively.<br>This study discovered that the phenylpropane metabolic pathway was an important step forward in understanding the stress resistance of highland barley, and provided new insights into the roles of molecular mechanisms in plant defense. |
| Order of Authors: | Zhenglian Xue |
| | Bingsheng Wang |
| | Changyu Qu |
| | Mengdie Tao |
| | Zhou Wang |
| | Guoqiang Zhang |

Powered by Editorial Manager® and ProduXion Manager® from Aries Systems Corporation

| | |
|---|---|
| | Shiguang Zhao |
| | Ming Zhao |
| Additional Information: | |
| Question | Response |
| Financial Disclosure<br><br>Enter a financial disclosure statement that describes the sources of funding for the work included in this submission. Review the submission guidelines for detailed requirements. View published research articles from PLOS ONE for specific examples.<br><br>This statement is required for submission and will appear in the published article if the submission is accepted. Please make sure it is accurate.<br><br>Unfunded studies<br>Enter: The author(s) received no specific funding for this work.<br><br>Funded studies<br>Enter a statement with the following details:<br>• Initials of the authors who received each award<br>• Grant numbers awarded to each author<br>• The full name of each funder<br>• URL of each funder website<br>• Did the sponsors or funders play any role in the study design, data collection and analysis, decision to publish, or preparation of the manuscript?<br>• NO - Include this sentence at the end of your statement: The funders had no role in study design, data collection and analysis, decision to publish, or preparation of the manuscript.<br>• YES - Specify the role(s) played. | This research was funded by a grant Tibet Key Research and Development Program Program (Program no. XZ202001ZY0040N), a grant (2022jc19) from Science and Technology Plan Project of Wuhu, and a grant (B2018-03) from Xuancheng Industrial Technology Research Institute Support Project.<br>The funders had no role in study design, data collection and analysis, decision to publish, or preparation of the manuscript. |

Powered by Editorial Manager® and ProduXion Manager® from Aries Systems Corporation

| | |
|---|---|
| * typeset | |
| Competing Interests<br><br>Use the instructions below to enter a competing interest statement for this submission. On behalf of all authors, disclose any competing interests that could be perceived to bias this work—acknowledging all financial support and any other relevant financial or nonfinancial competing interests.<br><br>This statement is required for submission and will appear in the published article if the submission is accepted. Please make sure it is accurate and that any funding sources listed in your Funding Information later in the submission form are also declared in your Financial Disclosure statement. | The authors have declared that no competing interests exist. |

| | |
|---|---|
| View published research articles from PLOS ONE for specific examples.<br><br>NO authors have competing interests<br><br>Enter: The authors have declared that no competing interests exist.<br><br>Authors with competing interests<br><br>Enter competing interest details beginning with this statement:<br><br>I have read the journal's policy and the authors of this manuscript have the following competing interests: [insert competing interests here]<br><br>* typeset | |

Powered by Editorial Manager® and ProduXion Manager® from Aries Systems Corporation

| | |
|---|---|
| Ethics Statement<br><br>Enter an ethics statement for this submission. This statement is required if the study involved:<br><br>• Human participants<br>• Human specimens or tissue<br>• Vertebrate animals or cephalopods<br>• Vertebrate embryos or tissues<br>• Field research<br><br>Write "N/A" if the submission does not require an ethics statement.<br><br>General guidance is provided below. Consult the submission guidelines for detailed instructions. Make sure that all information entered here is included in the Methods section of the manuscript. | N/A |

*Powered by Editorial Manager® and ProduXion Manager® from Aries Systems Corporation*

| | |
|---|---|
| Format for specific study types<br><br>Human Subject Research (involving human participants and/or tissue)<br>• Give the name of the institutional review board or ethics committee that approved the study<br>• Include the approval number and/or a statement indicating approval of this research<br>• Indicate the form of consent obtained (written/oral) or the reason that consent was not obtained (e.g. the data were analyzed anonymously)<br><br>Animal Research (involving vertebrate animals, embryos or tissues)<br>• Provide the name of the Institutional Animal Care and Use Committee (IACUC) or other relevant ethics board that reviewed the study protocol, and indicate whether they approved this research or granted a formal waiver of ethical approval<br>• Include an approval number if one was obtained<br>• If the study involved non-human primates, add additional details about animal welfare and steps taken to ameliorate suffering<br>• If anesthesia, euthanasia, or any kind of animal sacrifice is part of the study, include briefly which substances and/or methods were applied<br><br>Field Research<br><br>Include the following details if this study involves the collection of plant, animal, or other materials from a natural setting:<br>• Field permit number<br>• Name of the institution or relevant body that granted permission | |
| Data Availability<br><br>Authors are required to make all data underlying the findings described fully available, without restriction, and from the time of publication. PLOS allows rare exceptions to address legal and ethical concerns. See the PLOS Data Policy and FAQ for detailed information. | Yes - all data are fully available without restriction |

| | |
|---|---|
| A Data Availability Statement describing where the data can be found is required at submission. Your answers to this question constitute the Data Availability Statement and will be published in the article, if accepted.<br><br>Important: Stating ‘data available on request from the author’ is not sufficient. If your data are only available upon request, select ‘No’ for the first question and explain your exceptional situation in the text box.<br><br>Do the authors confirm that all data underlying the findings described in their manuscript are fully available without restriction? | |

| | |
|---|---|
| Describe where the data may be found in full sentences. If you are copying our sample text, replace any instances of XXX with the appropriate details.<br><br>• If the data are held or will be held in a public repository, include URLs, accession numbers or DOIs. If this information will only be available after acceptance, indicate this by ticking the box below. For example: All XXX files are available from the XXX database (accession number(s) XXX, XXX.).<br>• If the data are all contained within the manuscript and/or Supporting Information files, enter the following: All relevant data are within the manuscript and its Supporting Information files.<br>• If neither of these applies but you are able to provide details of access elsewhere, with or without limitations, please do so. For example:<br><br>Data cannot be shared publicly because of [XXX]. Data are available from the XXX Institutional Data Access / Ethics Committee (contact via XXX) for researchers who meet the criteria for access to confidential data.<br><br>The data underlying the results presented in the study are available from (include the name of the third party | All the RNA-seq data generated in this research was deposited in the Sequence Read Archive database (www.ncbi.nlm.nih.gov/geo, accessed on 16 Nov 2022) at NCBI (National Center for Biotechnology Information) under accession number: PRJNA902282. |

| | |
|---|---|
| and contact information or URL).<br>• This text is appropriate if the data are owned by a third party and authors do not have permission to share the data.<br><br>* typeset | |
| Additional data availability information: | |

Powered by Editorial Manager® and ProduXion Manager® from Aries Systems Corporation

Manuscript

Click here to access/download;Manuscript;Manuscript.docx

RESEARCH ARTICLE

# Response of stress resistance in highland barley (*Hordeum vulgare L. var. nudum*) through phenylpropane metabolic pathway

**Z.-L. Xue*, B.-S. Wang*, C.-Y. Qu, M.-D. Tao, Z. Wang, G.-Q. Zhang & M. Zhao, S.-G. Zhao**

Anhui Engineering Laboratory for Industrial Microbiology Molecular Breeding, College of Biological and Food Engineering, Anhui Polytechnic University, Wuhu 241000, China

Correspondence:

Ming Zhao, Anhui Engineering Laboratory for Industrial Microbiology Molecular Breeding, College of Biological and Food Engineering, Anhui Polytechnic University, Wuhu241000,China. E-mail: zhaom@mail.ahpu.edu.cn China.

Shi-Guang Zhao, Anhui Engineering Laboratory for Industrial Microbiology Molecular Breeding, College of Biological and Food Engineering, Anhui Polytechnic University, Wuhu 241000, China. E-mail:zhaoshiguang@ahpu.edu.cn

*These authors contributed equally to the manuscript.


**ABSTRACT:**

- Highland barley (*Hordeum vulgare L. var. nudum*) is a ~~kind of grain crop, which~~grain crop that grows on the plateau under poor and high salt conditions. Therefore, to cultivate high-quality highland barley varieties, it is necessary to study the molecular mechanism of strong resistance in highland barley, which has not been clearly explained.
- In this study, a high concentration of NaCl (240 mmol/L), simulating the unfavorable environment, was used to spray ~~to~~ the treated highland barley seeds. Transcriptomic analysis revealed that the expression of more than 8,000 genes in highland barley seed cells was significantly altered, suggesting that the metabolic landscape of the cells was deeply changed under salt stress. Through the KEGG analysis, the phenylpropane metabolic pathway was significantly up-regulated under salt stress, resulting in the accumulation of polyphenols, flavonoids, and lignin, the metabolites for improving the stress resistance of highland barley seed cells, being increased 2.71, 1.22, and 1.17 times, respectively.
- This study discovered that the phenylpropane metabolic pathway was a~~n import~~ significant step forward in understanding the stress resistance of highland barley, and provided new insights into the roles of molecular mechanisms in plant defense.



**Keywords:**

Highland barley; salt stress; transcriptome; Phenylpropane metabolic pathway~~.~~

## INTRODUCTION

Highland barley (*Hordeum vulgare L. var. nudum*), barley genus (Obadi *et al.*, 2021), is mainly distributed in ~~the highland areas of northwest and southwest of China~~northwest and southwest China's highland areas. It is not only the main grain for Tibetan residents in China, but also the ~~main~~ primary raw material for ~~the production of~~producing beer, medicine, and health products (Zeng *et al.*, 2018). Compared with conventional barley varieties, highland barley has the properties of high protein, high dietary fiber, and low fat structure (Idehen *et al., 2017*), which meet the requirements of modern people for food. Therefore, highland barley has a broad application prospect in the functional food processing industry. Presently, research on highland barley mainly focuses on determining nutritional composition, extracting active ingredients, and analysing medical function (Goudar *et al.*, 2020; Nie *et al.*, 2022). For example, Dang (2022) studied the content and antioxidant capacity of β-glucan, melatonin, total phenols and flavonoids in highland barley seed during the germination stage~~,~~ and analyzed the antioxidant capacity of different tissues of highland barley. Therefore, the study of the resistance of highland barley to the ~~special~~ unique living environment is ~~very import~~ significant for ~~the cultivation of high-quality highland barley varieties and the improvement of~~cultivating high-quality highland barley varieties and improving the resistance of different plant species.

Salt stress is an important means of studying the stress resistance of plants (Mansour *et al.*, 2022). Soil salinization and soil osmotic pressure can be increased by the changes in the concentration of $Na^+$ and $K^+$, thus causing physiological drought and inhibiting normal plants growth (Ali *et al.*, 2019). Excessive salt ions accumulate in the plant tissues, resulting in ion antagonism, obstruction and destruction of normal physiological metabolism, and plant malformation or death (Tan *et al.*, 2019). In

addition, the redox balance in plant cells is destroyed under salt stress. Therefore, to effectively reduce the damage of reactive oxygen species, cells need to regulate the expression of a protective enzyme system to form a self-defense system (Baral., *2019*). In recent years, omics technology has developed rapidly, and it is wildly used to excavate stress tolerance genes in plants, especially in the analysis of the molecular mechanism of salt tolerance of plant cells (Wei *et al.*, 2022; Guo *et al.*,2020; Pan *et al.*,2022). However, the results of the molecular mechanism of salt stress in highland barley are still unsatisfactory (İbrahimova *et al.*, 2021). Therefore, the analysis of the molecular mechanism of salt stress in highland barley is very important for the research and cultivation ofresearching and cultivating plant varieties with high high-stress resistance.

Researchers mainly use transcriptomic strategies to study the differences in gene expression levels of highland barley under salt stress to excavate stress-related genes. For example, Lai (2020) utilized transcriptomics and proteomics strategies to analyze the molecular mechanism of salt stress response in highland barley seed cells during germination stage. They found that highland barley seeds could germinate normally under salt stress conditions and detected a large number ofmany genes related to salt response, which initially revealed the molecular mechanism of highland barley salt tolerance. However, the mechanism of salt stress response in related pathways remains unclear. Therefore, it is urgent to further investigate investigate further the molecular mechanism of salt stress in highland barley. Recently, the phenylpropane metabolic pathway, which produces polyphenols, flavonoids, lignin, and other secondary metabolites, has been reported as an important branch for the regulation of stress resistance in Pontederia cordata (Xin *et al.*, 2022), Walnut (Huang *et al.*, 2022), and watermelons (Zheng *et al.*, 2021). These metabolites can effectively decrease the

accumulation of reactive oxygen in plant cells and ~~avoid the destruction of cells morphology, structure, and physiological metabolic fun~~prevent cell morphology, structure, and physiological metabolic function destruction. Therefore, the phenylpropane metabolic pathway is closely related to the stress resistance of highland barley [19]. In this study, NaCl was used to simulate the salt stress environment to stimulate the seeds of highland barley during the germination stage~~, and t~~. Different periods' transcriptomics were used to analyze the molecular mechanism of highland barley stress resistance (Figure S1). Interestingly, the phenylpropane metabolic pathway was the most ~~important~~ critical pathway associated with the stress resistance of highland barley, which provided a theoretical basis for studying the resistance characteristics of highland barley and other plant species and the cultivation of resistant strains.

## MATERIALS AND METHODS

### Materials

Tibet Qing 2000 was provided by the Academy of Agriculture and Animal Husbandry Sciences of Tibet Autonomous Region. Gallic acid standard, rutin standard, ferulic acid standard, caffeic acid standard, Folin-Ciocalteu's phenol, and lignin content detection kit were purchased from Sangon Biotech. RNA Easy Fast RNA extraction kit for plant tissue, FastKing cDNA first strand synthesis kit, and RealUniversal color fluorescent quantitative premix reagent were purchased from TIANGEN (Beijing). qRT-PCR primers were synthesized at GenScript (Nanjing).

### Plant Growth Conditions

Highland barley seeds full of grains and no mechanical damage were selected and soaked in 1 g/L NaClO for 5 min. Then, highland barley seeds were cleaned off and

soaked for 10 h at 25 °C. The soaked seeds were placed into the marked seedling boxes. The treated seeds were germinated and grown in the intelligent artificial climate chamber (relative humidity of 80%, 12 h light/12 h dark). Seeds were sprayed with 240 mmol/L of NaCl and sterilized water each dadaily to collect highland barley seedlings on 0, 1, 3, 5, and 7 days.

**Preparation of Highland Barley Extract Solution**

Referring to Aguilera's method with slight modifications (Aguilera *et al.*,2015), approximately 0.2 g of highland barley freeze-dried powder was weighed and added into 5 mL 50% methanol solution for shock mixing. The solution was left in darkness at 4 °C for 16—18 hours, and then ultrasonic crushed in the water bath for 30 min. Finally, the treated solution was centrifuged at 10 000 r/min for 15 min at 4 °C, and the supernatant was filtered through the 0.45 μm microporous filter membrane and stored in the brown flask.

**Determination of Total Polyphenol Content**

The gallic acid standard solution (10 mg of gallic acid standard was weighed and dissolved in distilled water to 50 mL) of 0, 10, 20, 30, 40, and 50 μL were added into the well plate, respectively, mixing with 10 μL Folin-phenol reagent or static reaction for 6 minutes. 100 μL of 7% (W/V) Na2CO3 was added to the solution, then distilled water was added, and the solution volume was adjusted to 290 μL. Absorbance was measured at 760 nm, and the standard curve was drawn. 50 μL highland barley extract solution was added into the well plate and analyzed with the above method.

**Determination of Total Flavonoids Content**

The rutin standard solution (10 mg of rutin standard was weighed and dissolved in

80% ethanol to 50 mL) of 0, 10, 20, 30, 40, and 50μL were added to the well plate, respectively, mixing with 20 μL of 5% (W/V) Na2CO3 solution for a static reaction for 6 min. Then, 200 μL 10% (W/V) Al2(NO3)3 was added for a static reaction response for 6 min.

200 μL 4% (W/V) NaOH was added in the end, and the mixture solution was reacted for 10 min at room temperature for 15 minutes at room temperature for 15 minutes in the dark. Absorption was measured at 510 nm, and the standard curve was drawn. 50 μL highland barley extract solution was added to the well plate and analyzed with the above method.

**Determination of Lignin Content**

The lignin content was determined according to the characteristic absorption peak at 280 nm after acetylation of the phenolic hydroxyl group in the lignin. The operation procedure followed the instructions of the lignin content detection kit.

**HPLC Analysis of Ferulic Acid and Caffeic Acid**

The content of ferulic acid in highland barley extract solution was determined by C18 (4.6 mm×250 mm, 5 μm) column with reference to (Arslan, (2017). The mobile phase was methanol (A): 0.1% (V/V) phosphoric acid solution (B) (20: 80). The detection wavelength was 320 nm; the flow rate was 1.0 mL/min, the column temperature was 30 °C and the sample size was 10 μL. Ferulic acid standard solution (5.01 mg of standard ferulic acid was dissolved in 10 mL of methanol solution. 1 mL of the above solution was added into a 100 mL beaker, then methanol was added to the scale line and mixed to obtain 5.01 μg/mL of ferulic acid solution) was diluted to various concentrations and t. Methanol was added to the scale line and mixed to obtain 5.01 μg/mL of ferulic acid solution) was diluted to various concentrations. Then the

standard curve was drawn according to peak area and peak time. The content of caffeic acid in highland barley extract solution was determined by C18 (4.6 mm×250 mm, 5 μm) column ~~with reference to~~ (Wang, ~~(~~2015). The mobile phase was acetonitrile (A): 0.1% phosphoric acid solution (B) (10: 90); detection wavelength was 320 nm; flow rate was 1.0 mL/min; column temperature was

35 °C; sample size was 20 μL. Caffeic acid standard solution (21.72 mg ferulic acid standard was dissolved in 10 mL methanol solution. 1 mL of the above solution was added into a 100 mL beaker~~, then m~~. Methanol was added to the scale line and mixed to obtain 21.72 μg/mL) it was diluted to various concentrations, and then the caffeic acid standard curve was drawn. Highland barley extract solution was injected after ~~being filtered~~filtering through a 0.22 μm microporous filter membrane.

**Transcriptomic Sequencing**

The germination and cultivation process of highland barley seeds were the same as above. Highland barley seedlings with even growth at 0, 1, 3, 5, and 7 d were snapfrozen in liquid nitrogen and stored at -80 °C. Plant RNA was extracted and reverse transcribed to obtain cDNA. These libraries were sequenced by paired end (PE) sequencing based on the Illumina sequencing platform of Boser Biotech at Personalbio (Shanghai, China). KEGG enrichment analysis of differential genes in transcriptome data was performed by clusterprofiler.

**qRT-PCR**

Total RNA was extracted from highland barley using the RNA Easy Fast RNA Extraction kit purchased from TIANGEN (Beijing) according to the manufacturer’s instructions. gapdh was used as the housekeeping gene, qRT-PCR was performed using a FastKing cDNA first strand synthesis kit and RealUniversal color fluorescent

quantitative premix reagent purchased from Tiangen (Beijing, China) according to the manufacturer's instructions. For each PCR reaction, 10 µL of 2xRealUniversal PreMix, 0.6 µL of forward and reverse primers (Table S3), 1.5 µL of cDNA sample, and 7.3 µL of RNase-Free ddH2O were mixed, and PCR conditions were as follows: 95 °C for 15 min, followed by 40 cycles of 95 °C for 10 s and 60 °C for 30 s. The result was analyzed with the 2−ΔΔCt method and averaged with triplicates.

## RESULTS

### Analysis of Antioxidants in Highland Barley Seeds under Salt Stress

Salt stress will cause plant cells to produce a large amount of free oxygen, resulting in oxidative damage to plant cells (Yang *et al.*, 2018). Polyphenols and flavonoids were demonstrated as strong antioxidants, which could effectively remove oxygen free radicals in plant cells, thus protecting cells from the damage of cell-free toxicity (Shen *et al.*, 2022). Therefore, in this study, the content of total polyphenols and flavonoids in highland barley seeds during the ~~process of seed germination stage under salt stress and non-salt stress environment was firstly~~seed germination stage under salt stress and non-salt stress environment was first detected. As shown in Figure 1a and 1b, the maximum accumulation of polyphenols and flavonoids was 8.38±0.36 mg/g and 1.982±0.08 mg/g on the third day, respectively, reaching 2.71 and 1.22 times ~~of~~ the accumulation of polyphenols and flavonoids in highland barley seed cells under normal conditions. These results illustrated that polyphenols and flavonoids content of highland barley seed cells could be efficiently increased under salt stress, thus resulting in the rapid removal of free oxygen in highland barley seed cells.

It is widely known that ferulic acid and caffeic acid ~~were~~ are two ~~kinds of main~~ primary polyphenols in the plant (Lubbers *et al.*, 2021; Sharma *et al.*, *2019*). Therefore, ~~the content of ferulic acid and caffeic acid~~ferulic acid and caffeic acid content in the

highland barley seeds was also detected. The results exhibited that the maximum accumulation of ferulic acid and caffeic acid was 12.22±1.35 μg/g and 4.69±0.95 μg/g on the third day, respectively, reaching 1.36 and 1.62 times ~~of~~ the accumulation of ferulic acid and caffeic acid in highland barley seed cells under non-salt environment. These results implied that ferulic acid and caffeic acid would probably be the most important contents in highland barley for resisting disadvantage environments.

Besides the antioxidants, physical factors would also enhance the mechanical strength of highland barley seeds, thus resulting in protection against the external environment. Plant tissues contain a large amount of lignin, which is an important component to maintain the extremely high hardness of plants and protect tissues from corrosion (Chun *et al.*, 2019). Therefore, ~~the accumulation of ligni~~lignin accumulation during germination of highland barley seed was also detected in this study. As shown in Figure 2, lignin content increased with the germination time of highland barley seed and reached the highest accumulation on the seventh day. In addition, lignin synthesis capacity was significantly improved under salt stress, reaching 378.64±0.91 mg/g on the seventh day, which was 1.17 times ~~of~~ the seed content without salt stress. These results indicated that highland barley seeds ~~may~~ might resist the external environment by enhancing lignin synthesis in the process of stress resistance.

**Transcriptomic Analysis of Gene Expression Levels of Highland Barley Seed Cells under Salt Stress**

Under salt stress treatment, the synthesis capacity of antioxidants polyphenols and flavonoids of highland barley seeds, as well as lignin, were significantly improved during the germination stage, meaning that the transcription level of genes for the regulation and biosynthesis of these metabolites was significantly changed under salt stress. To confirm this hypothesis, transcriptomic profiles of highland barley seeds

germinated on the third and fifth days under salt stress were evaluated in comparison withing on the third and fifth days under salt stress were evaluated compared to seeds under non-salt stress. As shown in Figure 3a, 3b, and Figure S2, the transcriptional patterns of seeds germinated under salt stress on the third and fifth days exhibited considerable differences from that of seeds germinated under non-salt stress, with 5,220 and 4,788 upregulated genes and 4,293 and 4,565 downregulated genes (|log2FoldChange|> 1; $p < 0.05$), respectively, among about 55,706 total genes. These data clearly demonstrated that the global metabolism of highland barley seed cells underwent tremendous changes under salt stress. Apart from that, to explore the relationship between the changes in genes expression and germination times, the transcriptional patterns of seeds germinated at 1, 3, 5, and 7 days were compared with seeds germinated at 0 days under salt stress. The results showed that more than 20% of the genes in highland barley seed cells were significantly altered during the germination stage under salt stress (Figure 3a and 3d). Except that, the number of up-regulated genes was the largest on the third day during the germination stage, the time at whichwhen the accumulation of antioxidants was the maximum. These results illustrated that the metabolism pathways of highland barley seed cells were significantly altered under salt stress, especially during the germination stage after 3 days.

To clearly confirm the relationship between the changed genes, the Venn diagram was usedhe Venn diagram was used to confirm the relationship between the changed genes. As exhibited in Figure 3c, the transcription levels of 4453 genes and 4295 genes were just changed on the third and fifth day, respectively, illustrating that the transcription levels of specific genes were differentspecific genes' transcription levels differed during different germination stages. Interestingly, the transcription level of about 5058 genes was significantly altered both on the third day and fifth day, indicating

that the transcription level of a large number of genes was continuously affected under salt stress.

## Changes Analysis of Metabolic Pathways of Highland Barley Seeds Cells under Salt Stress

After salt stress treatment, the transcription level of more than 20% of the genes in highland barley seed cells was changed. In addition, the accumulation of antioxidants, including polyphenols and flavonoids, and anticorrosive substance, lignin, of highland barley seeds were efficiently increased. Therefore, metabolic pathways in highland barley seed cells should be significantly changed. To investigate this hypothesis, KEGG (Kyoto Encyclopedia of Genes and Genomes) pathway enrichment analysis was used to identify metabolic differences between highland barley seed cells during the germination stage under salt and non-salt stress (Figure 4 and Figure S3). Changes in ribosomal pathways stood out among metabolic variations. Among a total of 123 genes involved in ribosomal pathways, more than 90 genes exhibited upregulated transcriptional levels on the third day during germination stage (Table S1), suggesting that profound changes occurred in the global gene translation networks of the highland barley seed cells under salt stress. Interestingly, significant changes took place in the phenylpropane biosynthesis pathways. Under salt stress, 122 genes and 48 genes in the phenylpropane biosynthesis pathway (total 351 genes) were efficiently upregulated and down-regulated, respectively, during the germination stage (3 day), thus resulting in the improvement of the accumulation of its metabolites, such as polyphenols, flavonoids, and other substances, in highland barley seeds. It is worth noting that significant changes also took place in the glycolysis and TCA cycle pathways. Interestingly, some key genes in the TCA cycle were significantly downregulated (3 genes up-regulated

and 3 genes down-regulated), while some key genes of the glycolysis pathway were upregulated (19 genes up-regulated and 22 genes down-regulated), indicating that additional precursors could be available for the biosynthesis above metabolites in highland barley seeds under salt stress.

The phenylpropanoid metabolic pathway is the core pathway for ~~the production of~~producing polyphenols, flavonoids, and other substances, which have been demonstrated as antioxidants in plants to defend against stress. This metabolic pathway starts from phenylalanine, ~~which is then~~ catalyzed by phenylalanine ammonia lyase (PAL) to produce trans-cinnamic acid. In addition, cinnamic acid 4-hydroxylase (C4H) catalyzes trans-cinnamic acid to produce coumaric acid, ferulic acid, erucic acid, caffeic acid, and others (Wu *et al.*, 2021; Luo *et al.*, 2022). Finally, 4-coumarate-CoA ligase (4CL), cinnamyl-alcohol dehydrogenase (CAD), caffeate 3-O-methyltransferase (COMT), caffeoyl CoA 3-O-methyltransferase (COMT), caffeoyl CoA 3-O-methyltransferase (CCoAOMT), and cinnamoyl-CoA reductase (CCR) work together to synthesize phenylpropanoid metabolites, such as lignin, flavones, and polyphenols. In the analysis of KEGG data (Figure 5, Figure S4, and Table S2), we found that more than half of the genes related to the phenylpropane metabolic pathways were significantly up-regulated, while less than 10% were downregulated. These results indicated that highland barley seed cells enhanced the synthesis ability of polyphenolic metabolites via enhancing phenylpropane metabolic pathways under salt stress, thus reducing ~~the content of free oxygen~~free oxygen content in cells and defending stress resistance.

**Verification of the Key Genes in Phenylpropane Pathway**

In order to further verify the key points of the phenylpropane metabolic pathway in highland barley seed cells in ~~the~~ response to salt stress, quantitative reverse

transcription polymerase chain reaction (qRT-PCR) was performed to analyze the transcriptional level of each key metabolic gene, including two PAL genes (D1007_08519 and D1007_11280), CH4 gene (D1007_58538), 4CL gene (D1007_39853), COMT gene (D1007_58836), CCoAOMT gene (D1007_35630), CCR gene (D1007_03378), and CAD gene (D1007_24864). Comparison of the relative expression levels of these genes evaluated by FPKM and qPCR methods is shown in Figure 6. The results showed that the transcriptional levels of these 8 genes analyzed by qRT-PCR were consistent with that of RNA seq, which verified that the key points of the phenylpropane metabolic pathway were indeed significantly changed under salt stress.

## DISCUSSION

Highland barley is an important grain crop in the plateau area of China. It is rich in nutrients and meets people's demand for a healthy diet (Luo *et al.*, 2022). In addition, highland barley has a strong ~~resistance to stress~~stress resistance~~,~~ so as to ensures the normal growth of highland barley crops in the adverse environment (Obadi *et al.*, 2021). Therefore, in recent years, more and more researchers have begun to study the stress resistance mechanism of highland barley, which not only helps to cultivate highland barley crops suitable for different adverse environments~~,~~ but also provides a reference for the improvement of stress resistance of other crops. However, the current research on stress resistance in highland barley has mainly focused on the content analysis of metabolites (Bao *et al.*, 2022; Jin *et al.*, 2022; Yang *et al.*, 2018), and gene mining and verification through omic, the work for the identification of metabolic pathways related to stress resistance is unsatisfactory. ~~In this study, the germination of highland barley seeds under salt stress was used~~This study used the germination of highland barley seeds under salt stress to simulate the unfavorable environment of highland barley crops~~, and~~

t. The effective improvement of related genes in the phenylpropane metabolic pathway was located through the transcriptional level of highland barley seeds during different stages under salt stress.

The phenylpropane metabolic pathway is ubiquitous in plants, and its secondary metabolites play an important role in plant growth, development, and defending stress (Zhang *et al.*, 2022). The biosynthesis of phenylpropane metabolites starts from the common metabolic pathway of phenylpropane, and t. Several branches of phenylpropane metabolism arehere are several branches of phenylpropane metabolism downstream, including the lignin synthesis pathway, polyphenol synthesis pathway, and flavonoid synthesis pathway. Lignin is the main component of the cell wall (Hu *et al.*, 2022) that supports the plant upright and allows the xylem to withstand the negative pressure generated during water transport. Under salt stress, the lignification degree of plant root cell wall will increase, which can not only effectively prevent the internal ion absorption of cells, but also enhance the structural rigidity and firmness of conduction tissue and improve the plant salt tolerance (Zagorchev *et al.*, 2014). Polyphenols and flavonoids have been demonstrated as strong antioxidants, which can effectively removeeffectively removing oxygen free radicals in plant cells, thus participating in plant stress resistance. Therefore, the phenylpropane metabolic pathway is probably the most important metaod method for the stress resistance in highland barley.

The phenylpropane metabolic pathway has not been reported to be related to stress resistance in highland barley, but it has been studied in other crops (Hussain *et al.*, 2020). For example, Kiani (2021) found that flavonoids in Tartary buckwheat flowers increased significantly after 12 h under salt stress, which may indicate that anthocyanins were synthesized from the corresponding products of salt stress to resist the adverse effects of salt stress (Ren *et al.*, 2021). Benincasa (2021) found that under salt stress,

~~the content of polyphenols in rapeseed was increased, improving the resistance ability of plants~~the content of polyphenols in rapeseed was increased under salt stress, improving plants' resistance ability to salt stress. Therefore, in this study, the barley seeds were treated with 240 mmol/L NaCl, and the total polyphenols, flavonoids, and lignin in highland barley seed cells were measured. It was found that under salt stress, the yield of highland barley seeds polyphenols, flavonoids, and lignin increased by about 50%, 25%, and 20%, respectively (Figure 1 and 2). The results indicated that highland barley seed cells ~~can~~ could effectively increase the biosynthesis capacity of polyphenols, flavonoids, and lignin by enhancing the phenylpropane metabolism pathway under salt stress, thus improving the ability of cells to degrade free oxygen generated under adverse conditions and resistance to mechanical forces.

The first three catalytic reactions in the phenylpropane metabolic pathway were demonstrated to be the core reactions of the whole metabolic pathway (Xue *et al.*, 2020). PAL is the first reactive enzyme in the phenylpropane metabolism pathway~~,~~ and also a rate-limiting enzyme in catalyzing phenylpropane to cinnamic acid, which is the precursor for the synthesis of downstream secondary metabolites (Nomberg *et al.*, 2022). Sang (2019) found that the PAL activity was increased in wheat roots with the increase of $Na^+$, especially in the salt-sensitive ~~genotypes of wheat~~wheat genotypes. This study found that the transcription of three PAL genes (a total of 9 PAL genes) was significantly upregulated under salt stress conditions, particularly D1007_11280, with a maximum increase in gene transcription level (Log2FoldChange= 4.945). With the coordinated expression of C4H and PAL, p-hydroxycinnamic acid can be produced by hydroxylation of the C4 position of cinnamic acid, which is the second step of phenylpropane metabolism. Baek (2008) cloned C4H gene from black raspberry and found that it had a differential expression pattern during fruit development~~, and t~~. The

content of black raspberry flavonoids could be changed by the gene regulation of C4Hgene regulation of C4H could change the content of black raspberry flavonoids.

In this study, we found that the transcriptional level of one of the C4H genes, D1007_58538, in highland barley seed cells was significantly up-regulated, reaching about 8 (23.003) times on the third day during germination stage, respectively (Figure 5 and Table S2). 4CL is the last catalytic enzyme in the three common standard steps of the phenylpropane metabolic pathway, and it is also the main fulcrum enzyme in the secondary metabolism of the life-generating thioester (Lavhale *et al.*, 2018). Our study found that transcription levels of two 4CL genes (a total of three) in highland barley seed cells were significantly up-regulated (Table S2). Several key enzymes, including CCR, CAD, COMT, and CCoAOMT, work for the production ofto produce phenylpropane

secondary metabolites, such as polyphenols, flavonoids, and lignin. In this study, three CAD genes, four CCR genes, five COMT genes, and one CCoAOMT gene were demonstrated to respond to salt stress (Table S2). The results showed that transcription levels of these key enzymes reached the maximum on the third day during germination stage. It has been shown that the expression of these genes was considered as the regulatory factors controlling the phenylpropane pathway, and overexpression of these genes in highland barley would improve the resistance of highland barley to stress (Yadav *et al.*, 2020). CertainlyIndeed, the resistance mechanism of highland barley cannot focus on just one metabolic pathway. For example, it was found in this study that the glutathione synthesis pathway and energy synthesis pathway of highland barley seed cells were significantly improved after salt stress. These issues need to be further addressed in our future studies.

**ACKNOWLEDGEMENTS**

We gratefully acknowledge Personalbio (Shanghai, China) for technical assistance with the transcriptomic analysis.

**CONFLICT OF INTEREST**

The authors declare that they have no competing interests.

**AUTHOR CONTRIBUTIONS**

Conceptualization, Z.X, S.Z., B.W. and M.Z.; methodology, B.W. and M.Z.; software, S.Z., B.W., C.Q. and M.T.; validation, B.W., M.Z. and Z.W.; formal analysis, G.Z.; investigation, S.Z.; resources, S.Z.; data curation, S.Z.; writing—original draft preparation, B.W. and M.Z.; writing—review and editing, M.Z. and S.Z.; visualization, B.W.; supervision, S.Z.; project administration, Z.X., M.Z. and S.Z.; funding acquisition, Z.X., M.Z. and S.Z. All authors have read and agreed to the published version of the manuscript.

**Data Availability Statement**

All the RNA- seq data generated in this research was deposited in the Sequence Read Archive database (www.ncbi.nlm.nih.gov/geo, accessed on 16 Nov 2022) at NCBI (National Center for Biotechnology Information) under accession number: PRJNA902282.

**Funding**

This research was funded by a grant Tibet Key Research and Development Program Program (Program no. XZ202001ZY0040N), a grant (2022jc19) from Science and Technology Plan Project of Wuhu, and a grant (B2018-03) from Xuancheng Industrial Technology Research Institute Support Project.

## SUPPLEMENTARY MATERIALS

Additional supporting information may be found online in theSupporting Information section at the end of the article.

**Figure S1:** Phenotypic characterization of highland barley;

**Figure S2:** Volcano Plot analysis of differentially expressed genes;

**Figure S3:** GO enrichment analysis of differentially expressed genes;

**Figure S4:** KEGG analysis of phenylpropane metabolic pathway;

**Table S1:** Genes altered significantly in transcription level;

**Table S2:** Transcriptional level analysis of key genes in the phenylpropane metabolic pathway;

**Table S3:** Primers used for qRT-PCR.

## REFERENCES

Ali A., Maggio A., Bressan R.A., Yun D.J. (2019) Role and Functional differences of HKT1-Type transporters in plants under salt stress. *International journal of molecular,* **20,** 1059.

Aguilera Y., Herrera T., Liébana R., Rebollo-Hernanz M., Sanchez-Puelles. C., MartínCabrejas M.A. (2015) Impact of melatonin enrichment during germination of legumes on bioactive compounds and antioxidant activity. *Journal of agricultural and food chemistry,* **63,** 7967—7974.

Arslan B.N., Küçük M., Akar Z. (2017) An on-line HPLC system for detection of antioxidant compounds in some plant extracts by comparing three different methods. *Journal of chromatography.B,* **1052,** 66—72.

Baral A. (2019) Strawberries under salt stress: ALA and ROS to the rescue. *Physiologia plantarum,* **167,** 2—4.

Bao L., Bao G., Zhang X., Qu Y., Guo J., Pan X. (2022) Short-term effects of combined freeze–thaw and saline–alkali stresses on the physiological response in highland barley (Hordeum vulgare). *Functional plant biology,* **49,** 970—979.

Benincasa P., Bravi E., Marconi O., Lutts S., Tosti G., Falcinelli B. (2021) Transgenerational effects of salt stress imposed to rapeseed (*Brassica napus var. oleifera Del.*) plants involve greater phenolic content and antioxidant activity in the edible sprouts obtained from offspring seeds. *Plants,* **10,** 932.

Baek M.H., Chung B.Y., Kim J.H. (2008) cDNA cloning and expression pattern of cinnamate-4-hydroxylase in the Korean black raspberry. *BMB reports,* **41,** 529—536.

Chun H.J., Baek D., Cho H.M. (2019) Lignin biosynthesis genes play critical roles in the adaptation of Arabidopsis plants to high-salt stress. *Plant signaling & behavior,* **14,** 1625697.

Dang B., Zhang W.G., Zhang J., Yang X.J., Xu H.D. (2022) Evaluation of nutritional components, phenolic composition, and antioxidant capacity of highland barley with different grain colors on the qinghai Tibet plateau. *Foods,* **11,** 2025.

Goudar G., Sharma P., Janghu S., Longvah T. (2020) Effect of processing on barley βglucan content, its molecular weight and extractability. *International journal of biological macromolecules,* **162,** 1204—1216.

Guo X., Wang Q., Liu Y., Zhang X., Zhang L., Fan S. (2020) Screening of salt stress responsive genes in brachypodium distachyon (L.) beauv. by transcriptome analysis. *Plants,* **9,** 1522.

Huang R., Zhou Y., Jin F. (2022) Metabolome and transcriptome profiling unveil the mechanisms of polyphenol synthesis in the developing endopleura of walnut (Juglans regia L.). *International journal of molecular,* **23,** 6623.

Hu J., Zhuang Y., Li X. (2022) Time-series transcriptome comparison reveals the gene regulation network under salt stress in soybean (Glycine max) roots. *BMC plant biology,* **22,** 157.

Hussain K., Jaweed T.H., Kamble A.C. (2020) Modulation of phenylpropanoid and lignin biosynthetic pathway is crucial for conferring resistance in pigeon pea against Fusarium wilt. *Gene,* **851,** 146994.

Idehen E., Tang Y., Sang S. (2017) Bioactive phytochemicals in barley. *Journal of food and drug analysis,* **25,** 148—161.

İbrahimova U., Kumari P., Yadav S. (2021) Progress in understanding salt stress response in plants using biotechnological tools. *Journal of biotechnology,* **329,** 180—191.

Jin H.M., Dang B., Zhang W.G., Zheng W.C., Yang X.J. (2022) Polyphenol and anthocyanin composition and Activity of highland barley with different colors. *Molecules,* **27,** 3411.

Kim J.I., Dolan W.L., Anderson N.A., Chapple C. (2015) Indole glucosinolate biosynthesis limits phenylpropanoid accumulation in arabidopsis thaliana. *Plant Cell,* **27,** 1529—1546.

Kiani R., Arzani A., Mirmohammady Maibody S.A.M. (2021) Polyphenols, flavonoids, and antioxidant activity involved in salt tolerance in wheat, aegilops cylindrica and their Amphidiploids. *Frontiers in plant science,* **12,** 646221.

Lai Y., Zhang D., Wang J. (2020) Integrative transcriptomic and proteomic analyses of molecular mechanism responding to salt stress during seed germination in hulless barley.*International journal of molecular,* **21,** 359.

Lubbers R.J.M., Dilokpimol A., Visser J., de Vries R.P. (2021) Aspergillus niger uses the peroxisomal CoA-dependent β-oxidative genes to degrade the

hydroxycinnamic acids caffeic acid, ferulic acid, and p-coumaric acid. *Applied microbiology and biotechnology,* **105,** 4199—4211.

Luo F., Yan P., Xie L. (2022) Molecular mechanisms of phenylpropane-synthesisrelated genes regulating the shoot blight resistance of bambusa pervariabilis × dendrocalamopsis grandis. *International journal of molecular,* **23,** 6760.

Lavhale S.G., Kalunke R.M., Giri A.P. (2018) Structural, functional and evolutionary diversity of 4-coumarate-CoA ligase in plants. *Planta,* **248,** 1063—1078.

Mansour M.M.F., Hassan F.A.S. (2022) How salt stress-responsive proteins regulate plant adaptation to saline conditions. *Plant molecular biology,* **108,** 175—224.

Nie C., Li T., Fan,M. (2022) Polyphenols in highland barley tea inhibit the production of advanced glycosylation end-products and alleviate the skeletal muscle damage.*Molecular nutrition & food research,* **66**, 2200225.

Nomberg G., Marinov O., Arya GC., Manasherova E., Cohen H. (2022) The Key Enzymes in the Suberin Biosynthetic Pathway in Plants: An Update. *Plants,* **11,** 392.

Obadi M., Sun J., Xu B. (2021) Highland barley: Chemical composition, bioactive compounds, health effects, and applications. *Food research international (Ottawa, Ont.),* **140,** 110065.

Obadi M., Sun J., Xu B. (2021) Highland barley starch (Qingke): Structures, properties, modifications, and applications. *International journal of biological macromolecules,* **185,** 725—738

Pan L., Ma J., Li J,, Yin B., Fu C. (2022) Advances of salt stress-responsive transcription factors in plants.*Sheng Wu Gong Cheng Xue Bao,* **38,** 50—65.

Ren T., Zheng P., Zhang K., Liao J., Xiong F., Shen Q., Ma Y., Fang W., Zhu X. (2021) Effects of GABA on the polyphenol accumulation and antioxidant activities in tea plants (*Camellia sinensis L.*) under heat-stress conditions. *Plant physiology and biochemistry,* **159,** 363—371.

Shen N., Wang T., Gan Q., Liu S., Wang L., Jin B. (2022) Plant flavonoids: classification, distribution, biosynthesis, and antioxidant activity. *Food chemistry,* **383,** 132531.

Sharma A., Shahzad B., Rehman A., Bhardwaj R., Landi M., Zheng B. (2019) Response of phenylpropanoid pathway and the role of polyphenols in plants under abiotic Stress. *Molecules,* **24,** 2452.

Sang S., Li S., Fan W., Wang N., Gao M., Wang Z. (2019) Zinc thiazole enhances defense enzyme activities and increases pathogen resistance to Ralstonia solanacearum in peanut (*Arachis hypogaea*) under salt stress. *PLoS One,* **14,** e0226951.

Tan X., Long W., Zeng L. (2019) M elatonin-induced transcriptome variation of rapeseed seedlings under salt stress. *International journal of molecular,* **20,** 5355.

Wei J., Liang J., Liu D., Liu Y., Liu G., Wei S. (2022) Melatonin-induced physiology and transcriptome changes in banana seedlings under salt stress conditions. *Frontiers in plant science,* **13,** 938262.

Wu J., Niu Z., Lu X. (2021) Transcriptome in combination proteome unveils the phenylpropane pathway involved in garlic (Allium sativum) greening. *Frontiers in nutrition,* **8,** 764133.

Wang J., Zhao Y.M., Zhang M.L., Shi Q.W. (2015) Simultaneous determination of chlorogenic acid, caffeic acid, alantolactone and isoalantolactone in Inula helenium by HPLC. *Journal of chromatographic science,* **53,** 526—530.

Xin J., Zhao C., Li Y., Ma S., Tian R. (2020) Transcriptional, secondary metabolic, and antioxidative investigations elucidate the rapid response mechanism of Pontederia cordata to cadmium. *Ecotoxicology and environmental safety,* **232,** 113236.

Xue J.S., Zhang B., Zhan H. (2020) Phenylpropanoid derivatives are essential components of sporopollenin in vascular plants. *Molecular plant,* **13,** 1644—1653.

Yang, Y.; Guo, Y. Unraveling salt stress signaling in plants. *J. Integr. Journal of integrative plant biology,* **2018,** *60,* 796—804.

Yang X.J., Dang B., Fan M.T. (2018) Free and bound phenolic compound content and antioxidant activity of different cultivated blue highland barley varieties from the Qinghai-Tibet plateau. *Molecules,* **23,** 879.

Yadav V., Wang Z., Wei, C. (2020) Phenylpropanoid pathway engineering: an emerging approach towards plant defense. *Pathogens,* **9,** 312.

Zeng X., Guo Y., Xu Q. (2018) Origin and evolution of qingke barley in Tibet. *Nature communications,* **9,** 5433.

Zheng Y., Xiao J., Zheng K., Ma J., He M., Li J., Li M. (2021) Transcriptome profiling reveals the effects of nitric oxide on the growth and physiological characteristics of watermelon under aluminum stress. *Genes,* **12,** 1735.

Zhang G., Yan Y., Zeng X., Wang Y., Zhang Y. (2022) Quantitative proteomics analysis reveals proteins associated with high melatonin content in barley seeds under NaCl-Induced salt stress. *Journal of agricultural and food chemistry,* **70,** 8492—8510.

Zagorchev L., Kamenova P., Odjakova M. (2014) The role of plant cell wall proteins in response to salt stress. *TheScientificWorldJournal,* **2014,** 764089.

**Figures and legends**

**Fig. 1.** Content analysis of highland barley seeds under salt stress. The contents of total polyphenols and total flavonoids changed on different germination days (0 day, 1 day, 3 day, 5 day, and 7 day) under salt stress. (**a**) The content of total polyphenols on different germination days. (**b**) The contents of total flavonoids changes on different germination days. (**c**) The contents of ferulic acid content on different germination days. (**d**) The contents of caffeic acid on different germination days. Error bars show the standard deviation from three independent experiments.

**Fig. 2.** Content analysis of lignin in highland barley seeds on different germination days. Error bars show the standard deviation from three independent experiments.

**Fig. 3.** Transcriptomic profiling of highland barley seeds cells under salt stress. (**a**) Number of genes significantly ($|\log_2 FoldChange| > 1$; $p < 0.05$) differentially expressed in highland barley seed cells during different times of germination stage under salt stress and non-salt stress. (**b**) Heatmap of transcript diversity across highland barley seeds cells during different germination stages (1 day, 3 day, 5 day, and 7 day) under salt stress. (**c**) Venn diagram analysis of highland barley seeds cells during the germination stage (3 day and 5 day) under salt stress and non-salt stress. (**d**) Heatmap of transcript diversity across highland barley seeds cells during the germination stage (3 day and 5 day) under salt stress and non-salt stress. Three biological replicates were performed per sample.

**Fig. 4.** Functional enrichment analysis of transcripts based on KEGG pathways. Three biological replicates were performed per condition.

**Fig.5.** Functional enrichment analysis of phenylpropanoid metabolic pathway. Red and green lines represent the upregulated and downregulated pathways, respectively.

**Fig. 6.** qRT-PCR verification of the key genes in the phenylpropane metabolic pathway. The mRNA expression levels were normalized to the expression level of *gapdh*. Error bars show the standard deviation from three independent experiments.

Figure

Click here to access/download;Figure;picture.docx

**Fig. 1.**

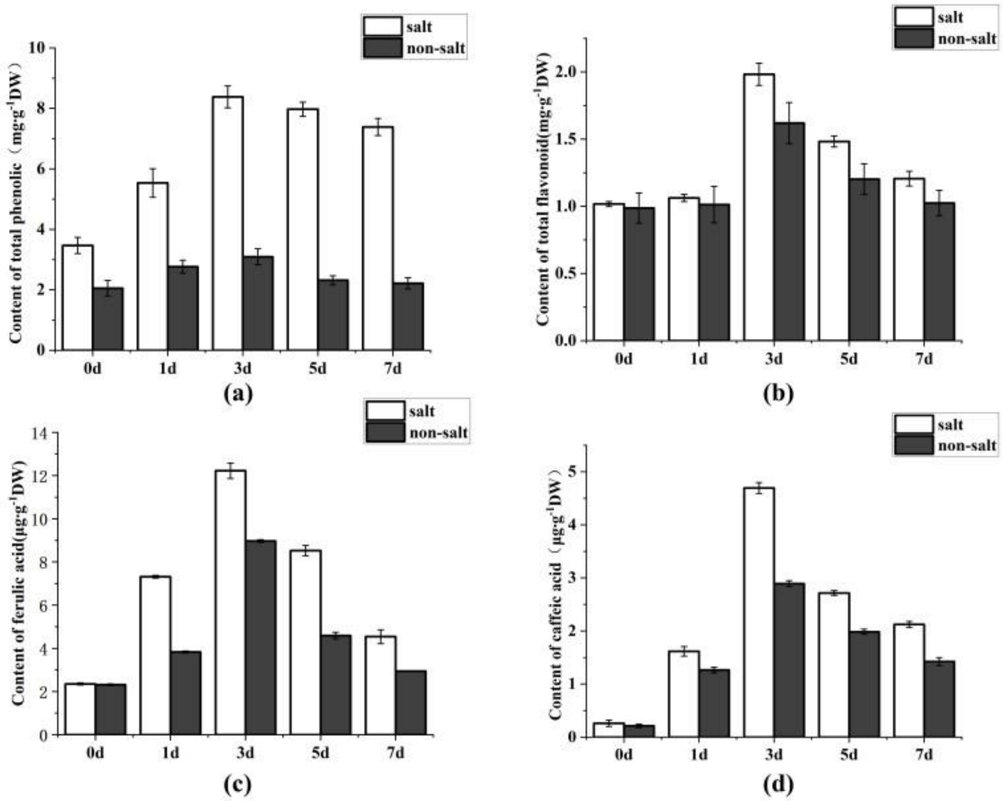

**Fig. 2.**

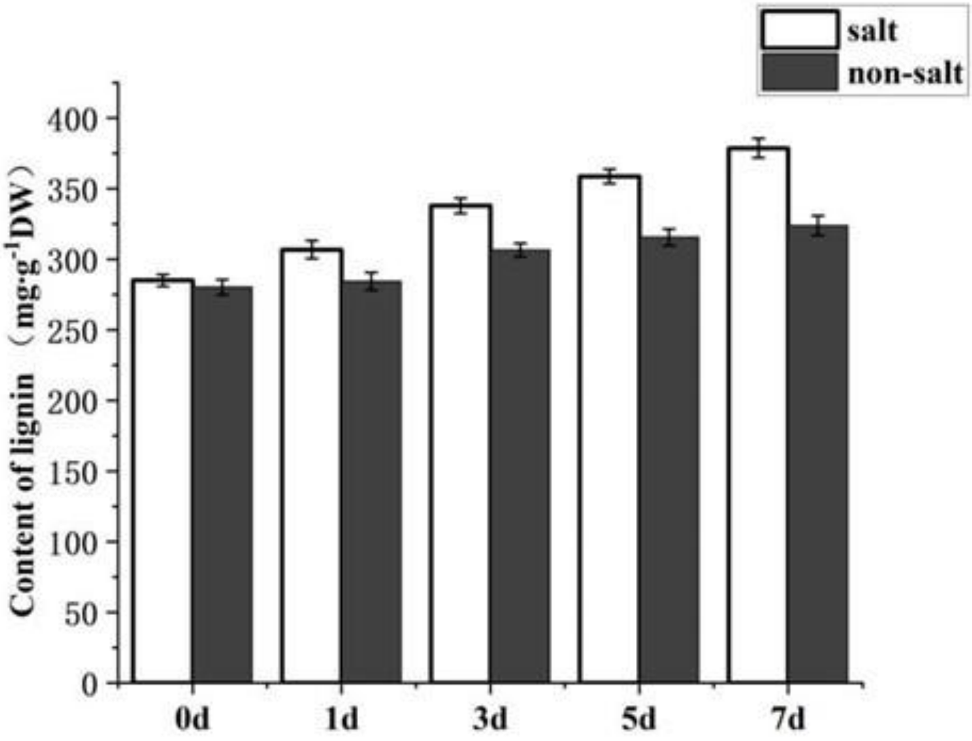

salt
non-salt
Content of lignin（mg·g-1DW）
400
350
300
250
200
150
100
50
0
0d
1d
3d
5d
7d

**Fig. 3.**

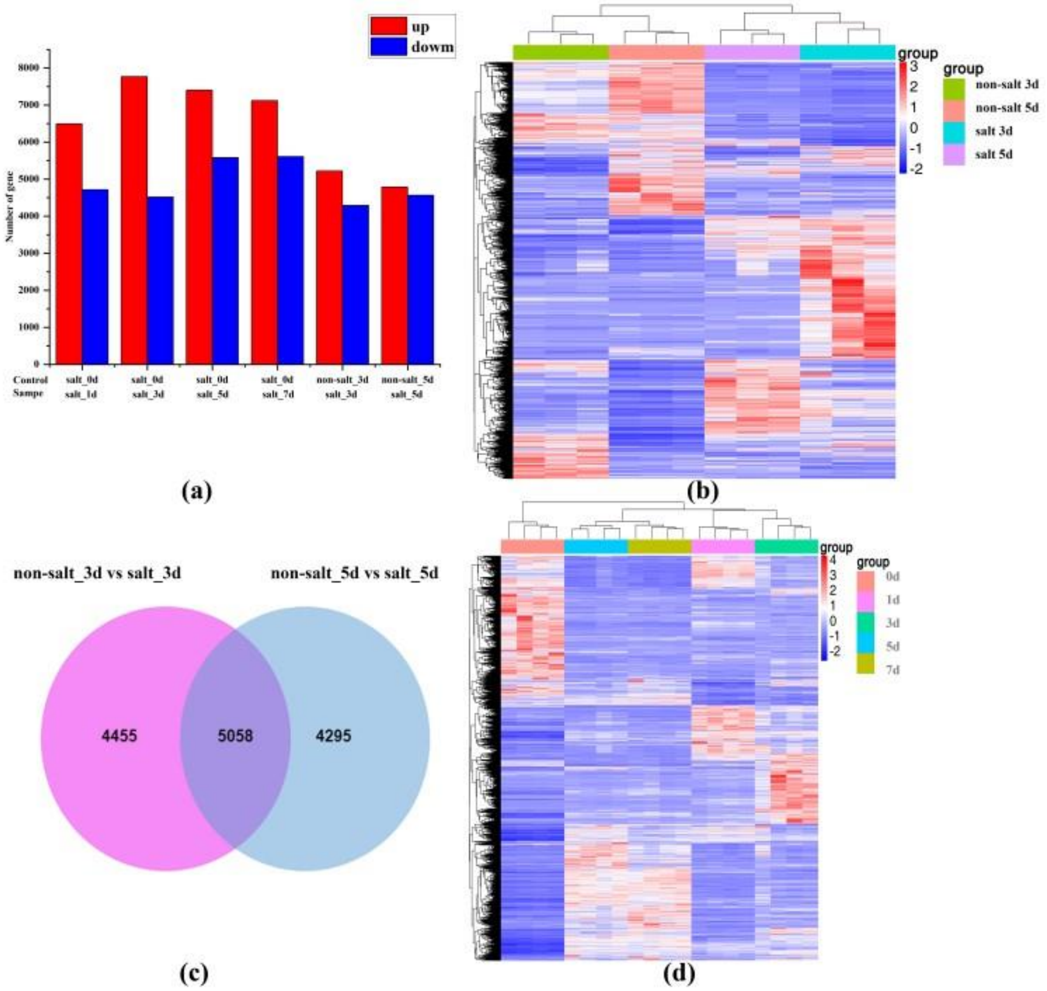
up
dowm
Number of gene
Control
Sampe
salt_0d
salt_1d
salt_0d
salt_3d
salt_0d
salt_5d
salt_0d
salt_7d
non-salt_3d
salt_3d
non-salt_5d
salt_5d
(a)
group
non-salt 3d
non-salt 5d
salt 3d
salt 5d
(b)
non-salt_3d vs salt_3d
non-salt_5d vs salt_5d
4455
5058
4295
(c)
group
0d
1d
3d
5d
7d
(d)

**Fig. 4.**

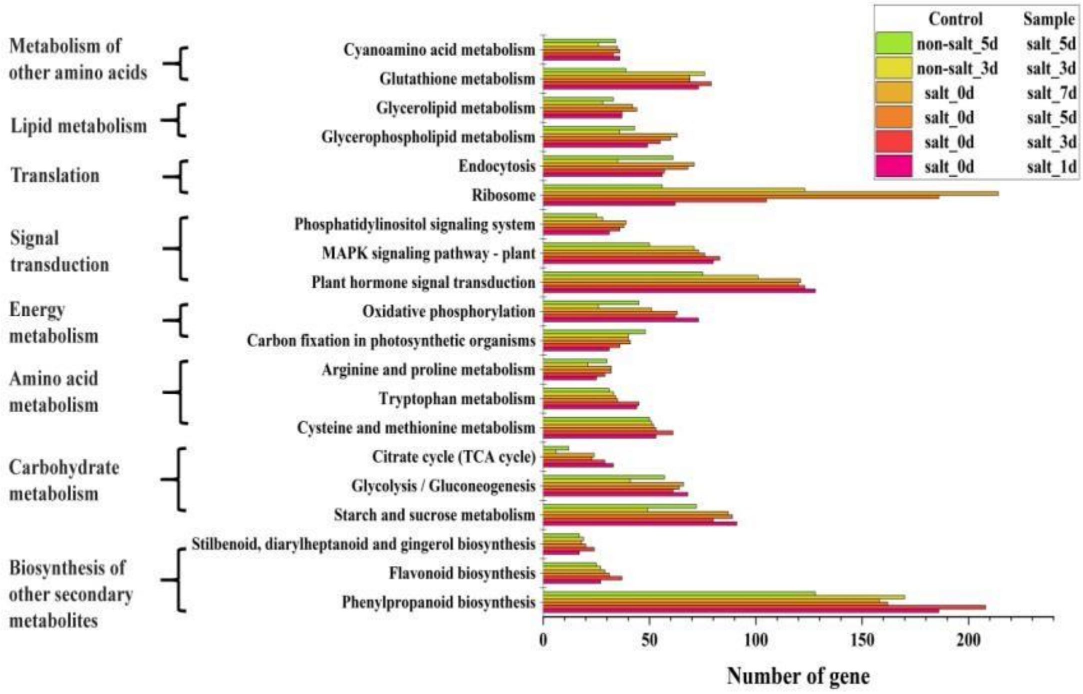
Metabolism of other amino acids
Lipid metabolism
Translation
Signal transduction
Energy metabolism
Amino acid metabolism
Carbohydrate metabolism
Biosynthesis of other secondary metabolites
Cyanoamino acid metabolism
Glutathione metabolism
Glycerolipid metabolism
Glycerophospholipid metabolism
Endocytosis
Ribosome
Phosphatidylinositol signaling system
MAPK signaling pathway - plant
Plant hormone signal transduction
Oxidative phosphorylation
Carbon fixation in photosynthetic organisms
Arginine and proline metabolism
Tryptophan metabolism
Cysteine and methionine metabolism
Citrate cycle (TCA cycle)
Glycolysis / Gluconeogenesis
Starch and sucrose metabolism
Stilbenoid, diarylheptanoid and gingerol biosynthesis
Flavonoid biosynthesis
Phenylpropanoid biosynthesis
0
50
100
150
200
Number of gene
Control
Sample
non-salt_5d salt_5d
non-salt_3d salt_3d
salt_0d salt_7d
salt_0d salt_5d
salt_0d salt_3d
salt_0d salt_1d

**Fig. 5.**

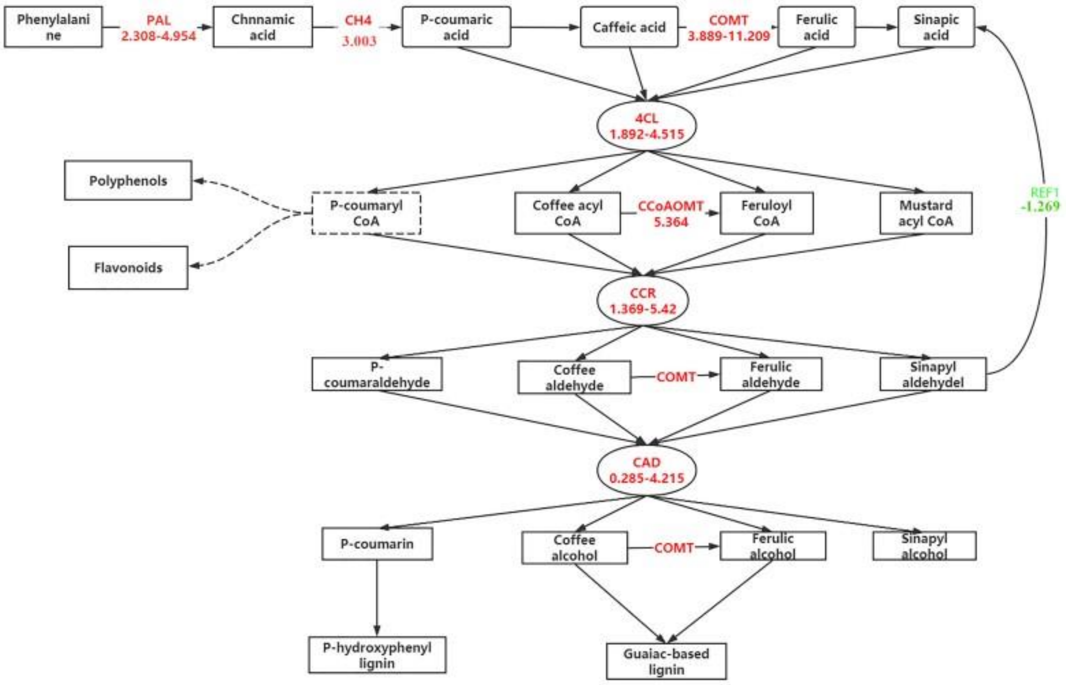

Phenylalanine
PAL
2.308-4.954
Chnnamic acid
CH4
3.003
P-coumaric acid
Caffeic acid
COMT
3.889-11.209
Ferulic acid
Sinapic acid
4CL
1.892-4.515
Polyphenols
Flavonoids
P-coumaryl CoA
Coffee acyl CoA
CCoAOMT
5.364
Feruloyl CoA
Mustard acyl CoA
REF1
-1.269
CCR
1.369-5.42
P-coumaraldehyde
Coffee aldehyde
COMT
Ferulic aldehyde
Sinapyl aldehydel
CAD
0.285-4.215
P-coumarin
Coffee alcohol
COMT
Ferulic alcohol
Sinapyl alcohol
P-hydroxyphenyl lignin
Guaiac-based lignin


**Fig. 6.**

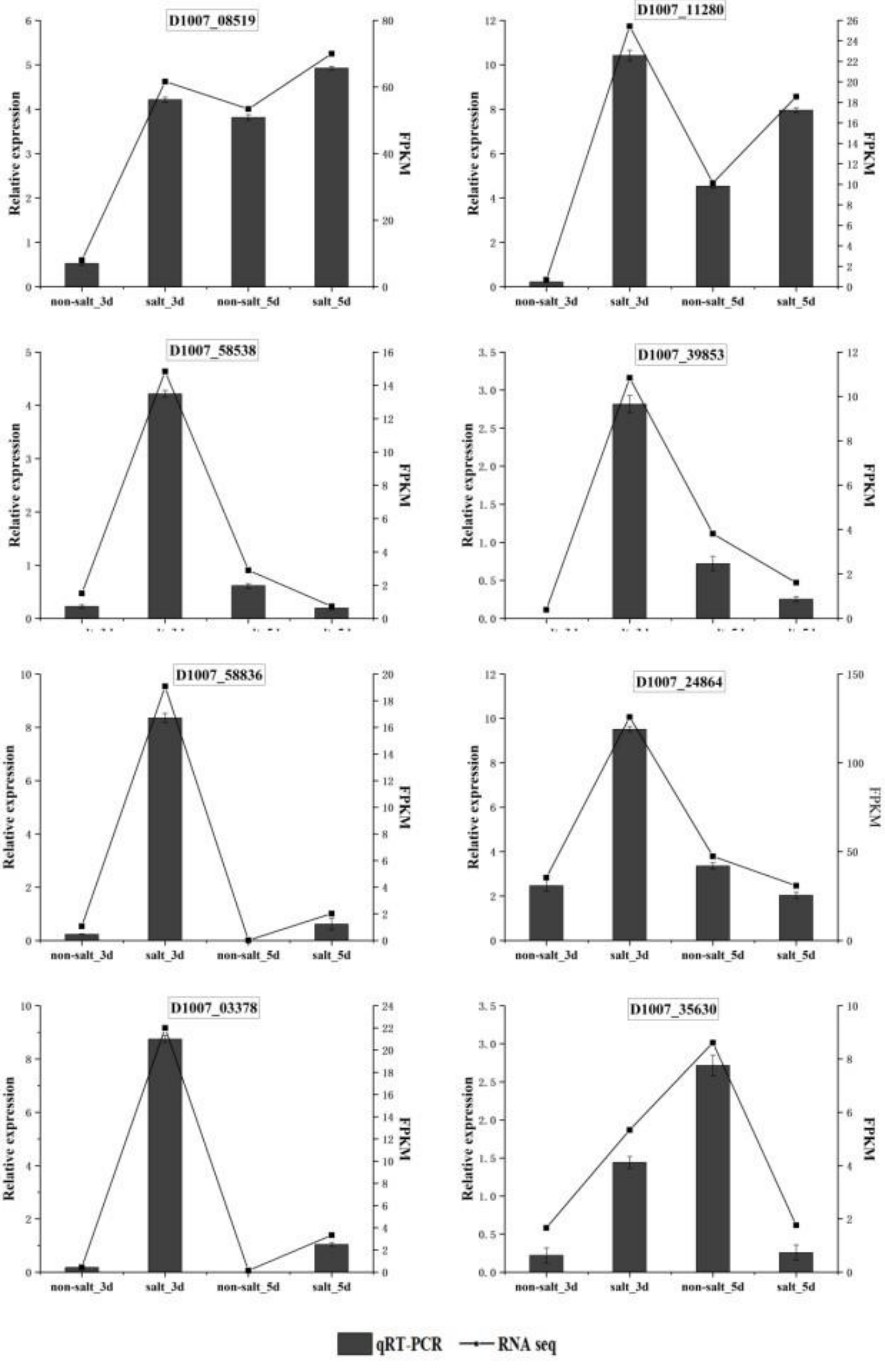

D1007_08519
D1007_11280
D1007_58538
D1007_39853
D1007_58836
D1007_24864
D1007_03378
D1007_35630
Relative expression
FPKM
non-salt_3d
salt_3d
non-salt_5d
salt_5d
qRT-PCR
RNA seq

Supporting Information

Click here to access/download
Supporting Information
Supplementary_information.docx
